# Profiles of pro-opiomelanocortin and encoded peptides, and their processing enzymes in equine pituitary pars intermedia dysfunction

**DOI:** 10.1371/journal.pone.0190796

**Published:** 2018-01-08

**Authors:** James L. Carmalt, Sima Mortazavi, Rebecca C. McOnie, Andrew L. Allen, Suraj Unniappan

**Affiliations:** 1 Department of Large Animal Clinical Sciences, Western College of Veterinary Medicine, University of Saskatchewan, Saskatoon, SK, Canada; 2 Department of Veterinary Biomedical Sciences, Laboratory of Integrative Neuroendocrinology, Western College of Veterinary Medicine, University of Saskatchewan, Saskatoon, SK, Canada; 3 Department of Veterinary Pathology, Western College of Veterinary Medicine, University of Saskatchewan, Saskatoon, SK, Canada; Universite de Rouen, FRANCE

## Abstract

Equine pituitary pars intermedia dysfunction (PPID) is characterized by hyperplasia of the pars intermedia (PI) melanotrophs of the pituitary gland (PG), and increased production of proopiomelanocortin (POMC). POMC is cleaved by prohormone convertase 1 (PC1) to produce adrenocorticotropic hormone (ACTH), and further processing of ACTH by PC2 to produce alpha-melanocyte stimulating hormone (α-MSH) and corticotropin-like intermediate peptide (CLIP). High plasma ACTH concentrations in horses with PPID might be related to reduced conversion of ACTH to α-MSH by PCs. The hypothesis of this study was that PC1 and PC2 expression in the pituitary gland are altered in PPID, resulting in an abnormal relative abundance of POMC derived proteins. The objectives of this study were to identify the partial sequences of equine POMC, PC1, and PC2 mRNAs; and to determine whether the expression of POMC, PC1, and PC2 mRNAs in whole pituitary extracts, and POMC-protein in the cavernous sinus blood of horses are altered in PPID. We confirmed (RT-PCR and sequencing) that the partial sequences obtained match the corresponding regions of predicted equine POMC, PC1 and PC2 sequences. The expression (quantification by RT-qPCR) of POMC, PC1 and PC2 mRNAs were found upregulated in the pituitary of horses with PPID. Plasma (measured using RIA/ELISA) ACTH and α-MSH were elevated in PPID horses. These results indicate distinct differences in gene and protein expression of POMC and its intermediates, and processing enzymes in PPID. It provides evidence to support the notion that local, pituitary-specific inadequacies in prohormone processing likely contribute to equine PPID.

## Introduction

Equine pituitary pars intermedia dysfunction (PPID), also known as equine Cushing’s disease, is a common disease of horses older than 15 years. It is the most common endocrine disease of the equine species with reported prevalence between 14% and 30% in aged horses [1]. Horses with PPID suffer significant morbidity and chronically poor welfare from myriad clinical signs. Hypertrichosis, chronic recurrent laminitis and redistribution of body fat occur frequently, as does increased susceptibility to opportunistic infections of the skin and respiratory tract [1]. PPID is proposed to be a primary neurodegenerative disorder characterized by a lack of dopaminergic inhibition of the melanotrophs of the pars intermedia [2,3]. *In vitro* and *in vivo* studies in rats and sheep have shown that in the absence of dopamine, hyperplasia and hypertrophy of the melanotroph cell line occur [4,5]. In mice with dopamine type-2 (D2R) receptor deficiencies, the gene expression of proopiomelanocortin (POMC) and its associated prohormone convertases (PC1 and PC2) are upregulated [6]. This results in an overabundance of downstream POMC-peptides, such as adrenocorticotrophic hormone (ACTH) cleaved from POMC by PC1; and alpha-melanocyte stimulating hormone (α-MSH) and corticotrophin-like immune peptide (CLIP) cleaved from ACTH by PC2, being released into the systemic circulation. This theory is given additional support by the fact that clinical signs and endogenous ACTH levels normalize in the face of treatment with a dopamine agonist [7].

We hypothesized that PC1 and PC2 levels in the pituitary gland are altered in PPID, resulting in an abnormal relative abundance of POMC derived peptides. Our aims were to confirm the native cDNA sequences of equine POMC, PC1 and PC2; to quantify and compare the mRNA expression of POMC and PCs in normal and PPID horses; and to quantify the plasma concentrations of POMC, ACTH, and α-MSH in the blood from the ventral cavernous sinus of normal and PPID horses.

## Materials and methods

### Ethics statement

All research protocols were approved by the University of Saskatchewan’s Animal Research Ethics Board and adhered to the Canadian Council on Animal Care guidelines for humane animal use (Protocol # 2010–0154).

### Animals

The whole pituitary gland of 10 normal and 6 PPID horses, submitted to the Department of Veterinary Pathology at the Western College of Veterinary Medicine, University of Saskatchewan, were collected immediately post-mortem. The gland was diced into small pieces and snap frozen in liquid nitrogen. Samples were stored at -80°C until total RNA extraction. Normal horses comprised of 4 geldings, one stallion, and 5 mares with a mean age of 10.1 yrs. The PPID horse group, which exhibited the classical signs of disease (hypertrichosis, laminitis and fat redistribution), comprised of 2 geldings and 3 mares (1 sex was not recorded) with a mean age of 22 yrs. The age disparity between groups was a function of the age-related nature of PPID.

An additional 6 normal horses and 6 PPID horses were used for ventral cavernous sinus blood collection. Normal horses (5 mares and 1 gelding) had a mean age of 12.2 yrs, whereas the horses exhibiting signs of PPID (5 mares and 1 gelding) had a mean age of 24.3 yrs. Horses were obtained by private donation to the research program, with signed, informed consent provided by the owners. They were housed in an outside paddock with hay and water provided *ad-libitum*. Blood was collected from the ventral cavernous sinus of standing horses using techniques reported earlier [8]. Blood was placed into plastic tubes containing EDTA (1.8 mg/mL), stored on ice during the collection period and then centrifuged at 4000 rpm for 15 minutes at 4°C. Plasma was collected and frozen at -80°C, for further hormone and PC measurements.

### Total RNA extraction and cDNA synthesis

Total RNA was extracted from the whole pituitary samples using TRIzol RNA isolation reagent (Invitrogen Inc. Canada). The entire pituitary gland was diced and mixed to prevent including just one region, 1 mL TRIzol was added to 1 mg of diced pituitary tissue samples, and homogenized by bead disruption using Tissuelyser (Qigen, Canada) and incubated in room temperature for 5 minutes. Phase separation was performed by adding 200 μL chloroform to each sample. Samples were mixed thoroughly, and centrifuged for 15 minutes at 13000 rpm. Then, the aqueous phase was transferred for RNA precipitation. Isopropanol (500 μL) was added and centrifuged for 15 minutes at 13000 rpm. Pelleted RNA was washed with 1 mL of 75% ethanol and dissolved in pure RNase free water. The purity of extracted RNA was assessed by optical density absorption ratio (OD 260/280 nm) using Nanodrop (ND-2000c, Thermo Scientific Inc. Canada). One microgram of total RNA was used for cDNA synthesis using iScript cDNA synthesis kit (BioRad) as directed by the manufacturer (Bio-Rad, Canada). cDNA samples were stored at -20°C until RT-PCRs and RT-qPCRs.

#### Primers

Primers were designed using Primer-Blast^TM^ from National Center for Biotechnology Information (www.ncbi.nlm.nih.gov/tools/primer-blast/). *Equus caballus* POMC (GenBank: XM_014731265.1), PC1 (GenBank: XM_001504608.4), and PC2 (GenBank: XM_001491591.5) primer sets were designed based on partial predicted sequences with the accession numbers listed above. Nine internal control genes [9] were used for expression stability in the pituitary gland. These included glyceraldehyde 3-phosphate dehydrogenase (GAPDH), 18S rRNA (18S), beta- 2-microglobulin (B2M), beta actin (ACTB), succinate dehydrogenase complex (SDHA), beta glucoronidase (GUSB), and tyrosine 3-monooxygenase/tryptophan 5-monooxygenase activation protein (YWHAZ). Primer sequences are provided in Table 1. Primers were synthesized by IDT Inc. (Canada) and were validated and optimized for high primer efficiency and annealing temperatures. Then, primers were used for Reverse Transcription–Polymerase Chain Reaction (RT-PCR) or Reverse Transcription–Quantitative PCRs (RT-qPCR).

**Table 1:** Sequences of primers, gene bank accession number, amplicon size, and annealing temperature used for RT-PCR and RT-qPCR.

Gene	Primer	Accession No. GenBank	Amplicon Size (bp)	Annealing Temperature (°C)
POMC	F-CAAGATCGACCTCTCCGCTG*	XM_014731265.1	313 (389–702)	60
	R-GGTCACTTCCACATGGGTGC^*^			
	F-TCAGGCTTCTGTGGAAGT^+^			
	R-AAGTGGCCCATGACGTA^+^		178 (293–468)	61
PC1	F-TGGGTTGCTAAATGCCAAAGC*^+^	XM_001504608.4	300 (1535–1835)	60
	R-ACAACCGACTTTCTCTCGCC*^+^			
PC2	F-TGTGGGGTCGGAGTAGCATA*	XM_001491591.5	280 (1009–1289)	60
	R-CCGTCGATGCTGCTGACATT*			
	F-CAACGATCCCTATCCGTA^+^			
	R-CGTCATAAATGGCTGGTC^+^		168 (909–1077)	60
18S rRNA	F-AACGACACTCTGGCATGCTAACT*^+^	XM_001497064	98	60
	R-CGCCACTTGTCCCTCTAAGAA*^+^			
GAPDH	F-AGAAGGAGAAAGGCCCTCAG^+^	NM_001163856	87 (1088–1175)	56
	R-GGAAACTGTGGAGGTCAGGA^+^			
B2M	F-GTGTTCCGAAGGTTCAGGTT^+^	NM_001082502	102(98–200)	55
	R-ATTTCAATCTCAGGCGGATG^+^			
ACTB	F-CGACATCCGTAAGGACCTGT^+^	NM_001081838	99 (861–960)	56
	R-CAGGGCTGTGATCTCCTTCT^+^			
GUSB	F-GGGATTCGCACTGTGGCTGTCA^+^	XM_001493514.2	116 (784–900)	62
	R-CCAGTCAAAGCCCTTCCCTCGGA^+^			
SDHA	F-GCAGAAGAAGCCATTTGAGG^+^	XM_001490889.4	102 (2190–2292)	55
	R-CCTGTCGATTACGGGTCTGT^+^			
YWHAZ	F-TGTTGTAGGAGCCCGTAGGT^+^	XM_001492988	94 (443–537)	60
	R-ATTCTCGAGCCATCTGCTGT^+^			
SLC36A2	F-GCTTCTGCCACAGGCTTAAC^+^	XM_001501324.3	69 (332–401)	55
	R-CCGGCTTTGAGTCCATACAT^+^			
Cyp19a1	F-GAGATGCCGTGGGAATTCTAGC^+^	NM_001081805	109 (882–991)	55
	R-ACGTTTCTCAGCCAAAAT^+^			

*Used for RT-PCR

^+^Used for RT-qPCR

#### RT-PCR and sequencing

RT-PCRs were carried out using 1 μL of cDNA, 50 μM dNTP’s, 10x PCR reaction mix, 2.5 mM MgCl_2_ and 0.5 μL taq DNA polymerase (Invitrogen Inc. Canada) making total of 20 μL of reaction volume. PCR conditions were: initial denaturation for 5 minutes at 95°C followed by 35 cycles of denaturation at 95°C for 1 minute, annealing temperature (Table 1) and extension at 73°C for 1 minute, and a final extension of 73°C for 10 minutes. PCR products were electrophoresed on 1.5% agarose gel. The images of the gel were recorded under UV light using a GelDoc^TM^ EZ Imager (Bio-Rad, Canada). Bands of the expected size were excised from 1.5% agarose gel. Four bands, representing expected amplicons, were purified using QIAquick Gel extraction kit (Qiagen Inc. Canada) and sent for sequencing (NRC, Canada). Sequences obtained were analyzed by NCBI and aligned to predicted equine POMC, PC1, and PC2 using NCBI nucleotide blast tools (https://blast.ncbi.nlm.nih.gov/Blast) and the European molecular biology laboratory online tools (http://www.ebi.ac.uk/Tools/msa/clustalo/). We also performed a comparison of partial sequences from normal and PPID horses to determine whether there are any differences in the critical (mature peptide or enzyme) sites of the sequences. Once the sequences obtained, although partial, confirmed the predicted sequences, the same primers were used for RT-qPCRs.

#### RT-Quantitative PCR

cDNAs were amplified by CFX connect (Bio-Rad, Canada) using iQ SYBR green supermix (Bio-Rad, Canada), and forward and reverse primers shown in Table 1. For each sample, RT-qPCR was run in duplicate and repeated again to ensure consistency. The thermal profile for all reactions was 5 minutes at 95°C and 40 cycles of 10 seconds denaturation at 95°C, 30 seconds of optimum annealing temperature as shown in Table 1, and 10 seconds of final extension at 73°C. Specificity of amplified products in RT-qPCR assays were determined by analyzing the melting curve to differentiate the target amplicon from primer dimers and other nonspecific products. A single melt curve was observed for each primer set of all RT-qPCR reactions.

Our extensive search in the literature for a suitable internal control gene that was validated for use in pituitary samples from PPID horses was not successful. This required us to validate control genes that are unaltered in PPID. To determine a suitable internal control gene for use in the pituitary gland, genes previously validated for use in equine reproductive tissues [9] were examined (Table 1). Based on the results of this validation, the Ct values of POMC, PC1, and PC2 were normalized to YWHAZ, chosen as the housekeeping gene. Relative mRNA expressions of genes of interest were determined according to Livak and Schmittegen method [10]. All reagents were stored under desired condition until use.

### Hormone and enzyme measurements

Plasma ACTH concentrations were measured using a commercial sandwich assay (Siemens ACTH kit and Immulite 1000 Chemiluminescent System, Siemens Canada, Oakville, ON, Canada). The plasma samples and test controls (bi-level ACTH control module, Siemens Canada Oakville, ON, Canada), were thawed and maintained on ice until analysis. Intra-assay coefficients of variation were 4.61% to 0.33% for low and high control sera with mean ACTH concentrations of 31.45 or 460 pg/mL, respectively. The manufacturer’s stated analytical sensitivity, lower limit of detection, was 9 pg/mL. A competitive sandwich ELISA was performed for POMC (manufacturer’s stated analytical sensitivity was 0.1 ng/mL, intra-assay coefficients of variation were 11.19% to 11.48% for control sera with mean POMC concentrations of 0.39 or 8.08 ng/mL, respectively; MyBioSource Inc., San Diego, CA, USA; POMC- MBS012413) following manufacturers guidelines. A commercial RIA (Euria –α-MSH, Euro Diagnostica AB, Malmö, Sweden) was used for the determination of α-MSH concentrations (manufacturer’s stated analytical sensitivity was 5 pg/mL (3 pmol/L), intra-assay coefficients of variation were 0.81% to 5.52% for control sera with mean α-MSH concentrations of 20.5 pg/mL or 125.3 pg/mL (12.3 or 75.2 pmol/L), respectively). Manufacturer’s guidelines for the ACTH assay stated no cross-reactivity to α-MSH, but a 13–15% cross-reactivity to ACTH (18–39). Information contained with the α-MSH RIA states no cross-reactivity to ACTH.

### Statistical analysis

All values were reported as means ± SEM. Statistical analysis was performed using PRISM 5.0 (GraphPad Software, Inc. La Jolla, CA USA) and SPSS (SPSS version 21, IBM Canada Ltd, Markham, ON, Canada). Comparisons between groups were made using Student’s t-test (and a Mann–Whitney U test where appropriate. P values less than 0.05 were considered statistically significant.

## Results

### Identification of partial sequences of POMC, PC1, and PC2 mRNAs, and its expression in equine pituitary

**Fig 1:**
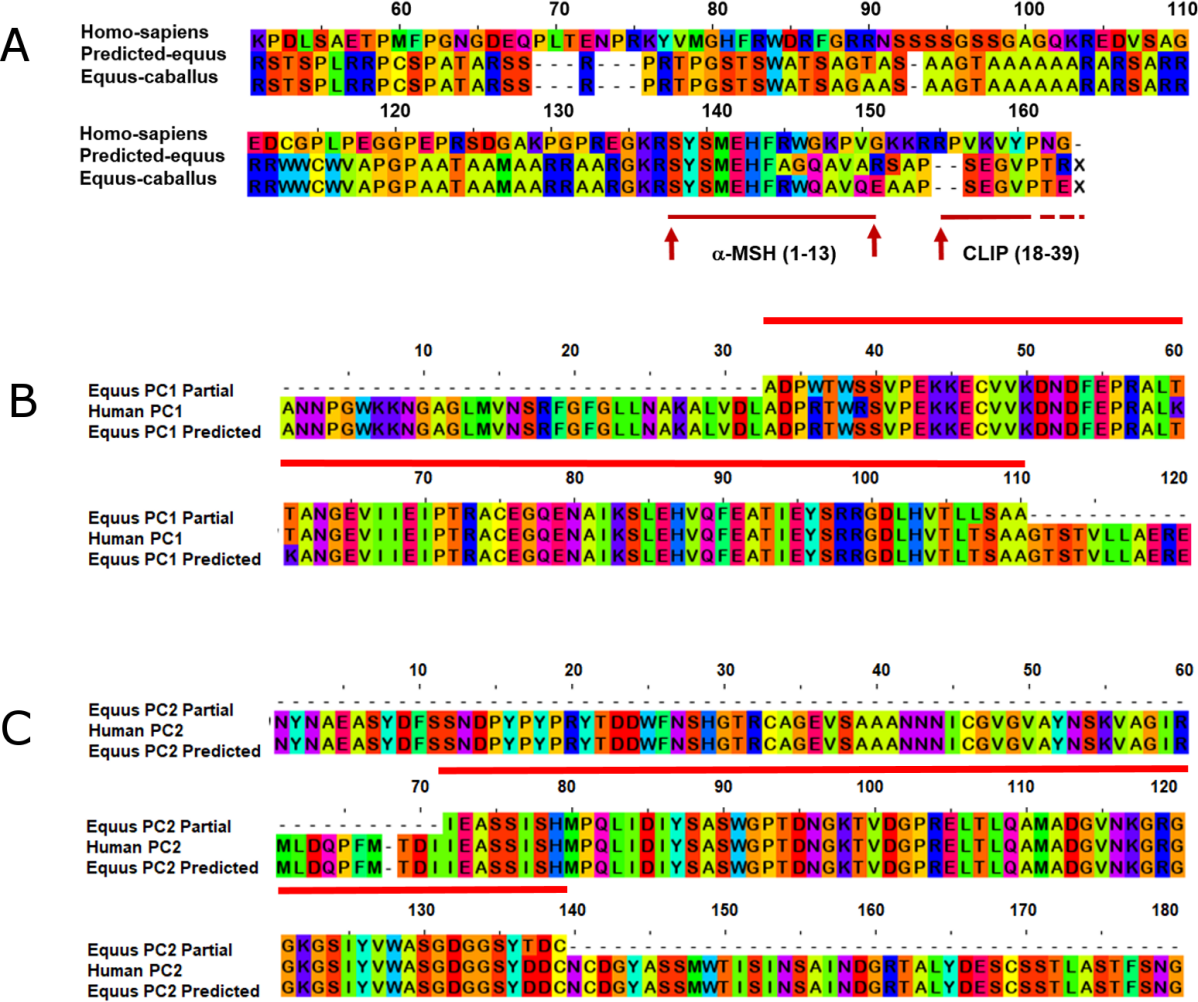
**Partial amino acid sequences for equine POMC (A), PC1 (B), and PC2 (C), compared to predicted *Equus caballus* and *Homo sapiens* sequences.** Red arrow (A) denotes the cleavage point within POMC for ACTH (the first 13 amino acids of which become α-MSH (red line), and the latter amino acids (18–39) subsequently become CLIP (red arrow with dashed end). Each amino acid is represented by a different color. PC1 and PC2 conserved regions are marked by a red line.

**Fig 2:**
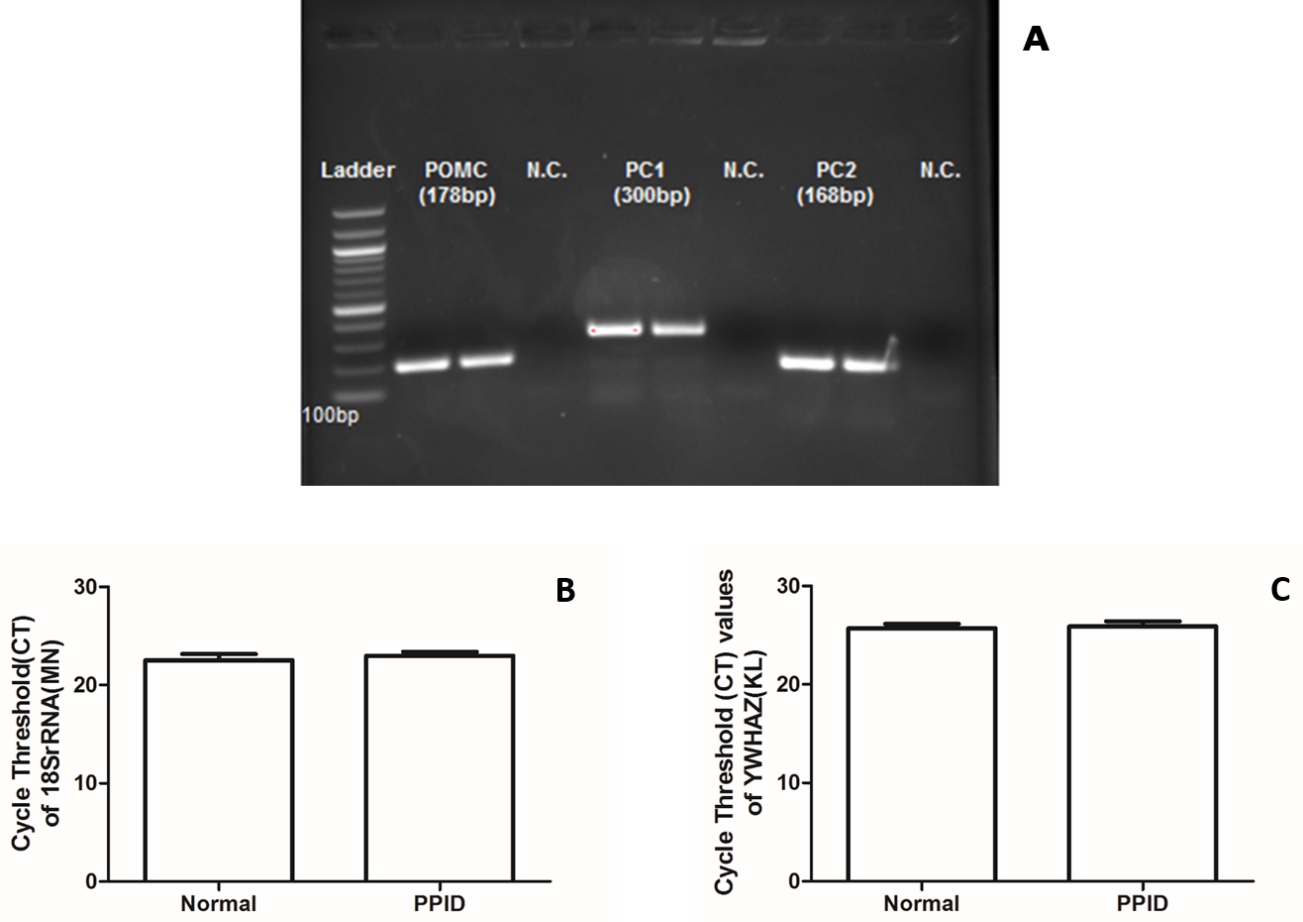
**A, A RT-PCR gel electrophoresis image showing the expression of equine POMC (178bp), PC1 (300bp) and PC2 (168bp) in the normal pituitary gland.** NC denotes no template control. **B,** Cycle threshold (Ct) values for the mRNA expression of 18S rRNA and YWHAZ (**C)** in the pituitary gland of normal (n = 10) and PPID (n = 6) horses. Bars denote mean values (+/- SEM).

We obtained partial mRNA sequences of POMC, PC1 and PC2, and these were found identical to the predicted equine sequences used to design the primers. It also confirmed the hormone-encoding region in the native equine POMC (GenBank Accession number: KY275178), and sequences of PC1 (GenBank Accession number: KY275179), and PC2 (GenBank Accession number: KY275180) mRNA sequences. *Equus* POMC partial nucleotide sequence exhibited high similarity to corresponding regions in the predicted horse (*Equus caballus* 95%), and human (*Homo sapiens*, 85%) POMC nucleotide sequences. The partial amino acid sequences of POMC, αMSH and CLIP (Fig 1A) were similar to predicted horse (*Equus caballus* 90%, 69.2%, and 91.9% respectively) and human (*Homo sapiens*, 80%; 69.2% αMSH; 10% CLIP) sequences Additionally, PC1 (Fig 1B) and PC2 (Fig 1C) partial sequences showed very high similarity to predicted horse (*Equus caballus*, 99% and 98%, respectively) and human (*Homo sapiens*, 98%, 96%, respectively) PC1 and PC2 nucleotide sequences. Horse PC1 and PC2 partial amino acid sequences were found to be almost identical to predicted horse (*Equus caballus* 99% and 100%, respectively) and human (98%, 100%, respectively) sequences. When comparing the amino acids sequences, we did not find any differences in POMC, PC1 or PC2 between normal horses and PPID horses. We also confirmed the expression of POMC, PC1 and PC2 mRNAs in the equine pituitary (Fig 2A). Of the 9 genes tested as internal control genes, 18S rRNA (Fig 2B) and YWHAZ (Fig 2C) genes were found to be most stable, and were used as internal controls genes for mRNA quantification studies. The remaining genes considered provided inconsistent Ct values, and were considered not reliable for use as housekeeping genes.

### POMC, PC1, and PC2 mRNA expression in the pituitary gland tissue

**Fig 3:**
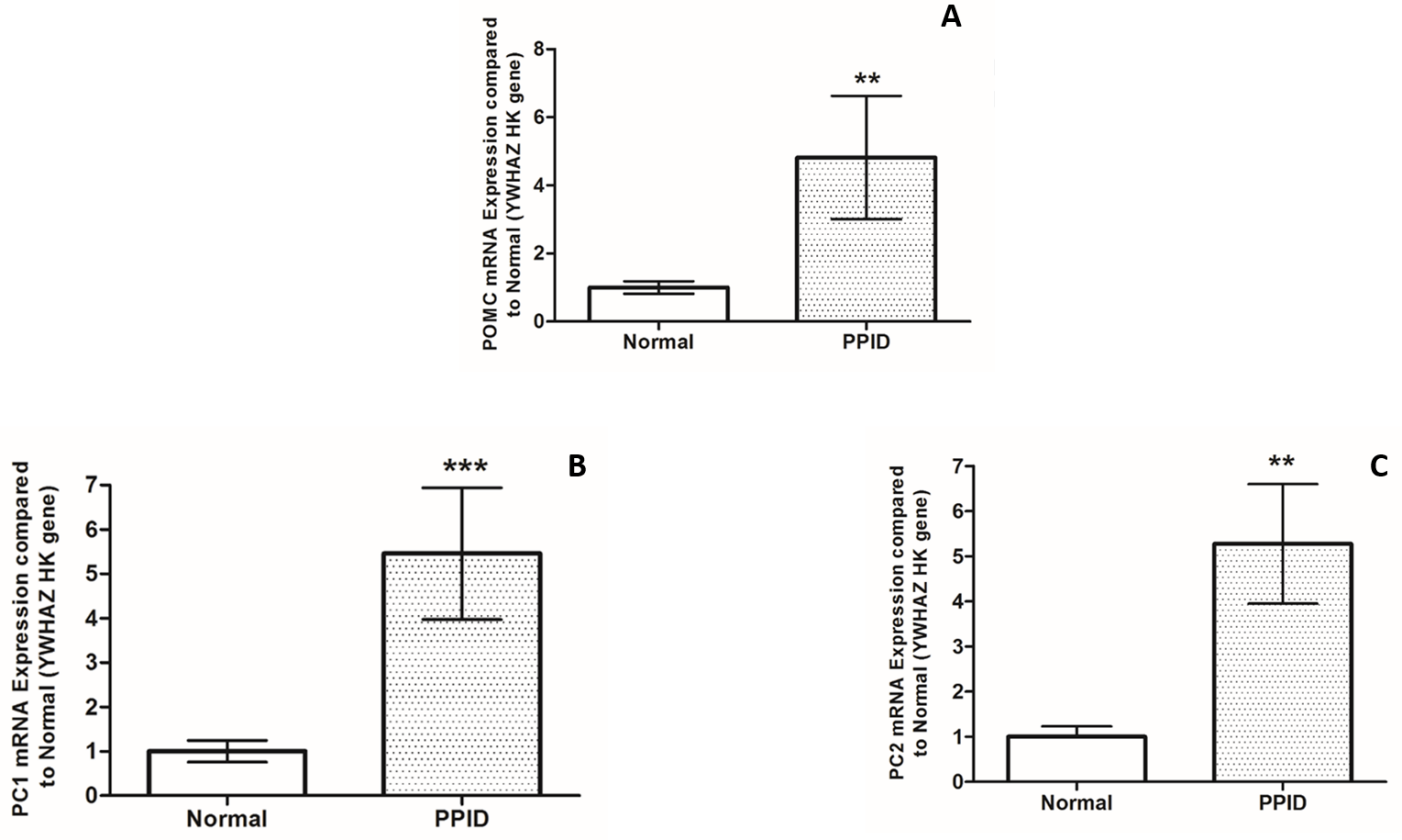
**POMC (A), PC1 (B), and PC2 (C) mRNA expression in the equine pituitary gland tissue from normal (n = 10) and PPID (n = 6) horses.** Bars denote mean values (+/- SEM). *** = p<0.001, ** = p<0.01.

POMC (4.8 fold increase, p = 0.0083, Fig 3A), PC1 (5.45 fold increase, p = 0.0007, Fig 3B), and PC2 (5.27 fold increase, p = 0.011, Fig 3C) mRNAs were significantly upregulated in the pituitary gland of horses with PPID (n = 6), when compared to normal horses (n = 10).

### POMC, ACTH, and α-MSH in plasma from normal and PPID horses

**Fig 4:**
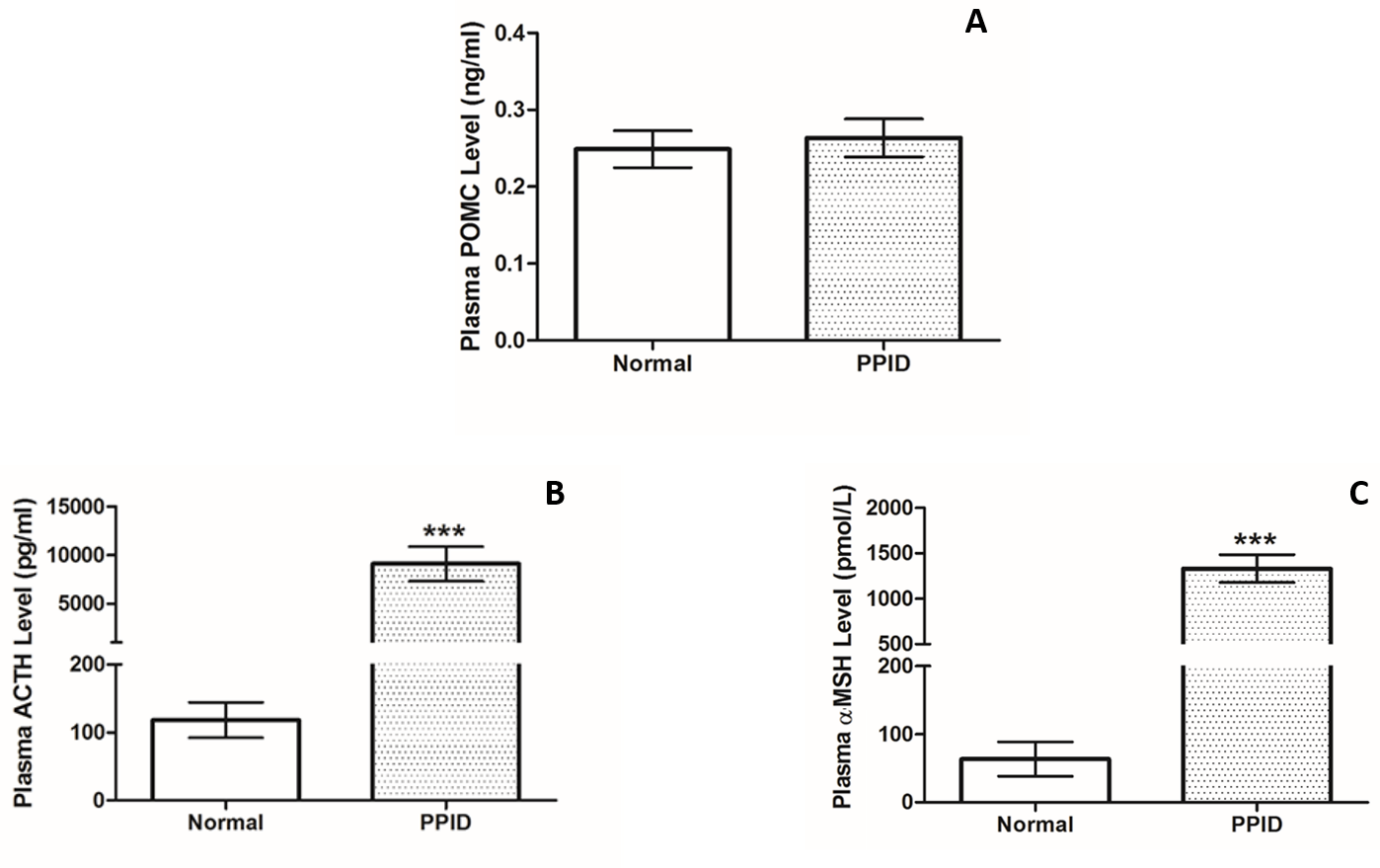
**Plasma concentrations of POMC (A), ACTH (B), and α-MSH (C), in normal (n = 6) and PPID (n = 6) horses.** Bars denote mean values (+/- SEM). *** = p<0.001.

There were no differences in POMC levels between normal (n = 6) and PPID horses (n = 6, p = 0.68, Fig 4A). ACTH (p = 0.0005, Fig 4B) and α-MSH (p = 0.004, Fig 4C) were significantly higher in PPID horses.

## Discussion

Our research presented here provides three sets of novel information in equine endocrinology. First, this study is the first to confirm the predicted equine POMC, PC1 and PC2 mRNA sequences, and found its expression of all three transcripts in the equine pituitary. Our sequencing data, although provided only partial sequences, matched that of the predicted sequences of all three genes. Additionally, within the sequences confirmed here, there are no key differences in the amino acid sequence of hormones encoded in POMC, or PC1 and PC2 between normal and PPID horses. While the identification of full-length sequences is critical, these highly conserved sequence fragments obtained here is important. For example, research in other species suggest that a significant portion of the elevated endogenous ACTH in PPID horses is likely not bioavailable due to sequence dissimilarities [7,11]. A mutation in the POMC gene was reported in humans, resulting in an immunologically active, but biologically inactive ACTH [12]. This mutation resulted in an ACTH analogue that could not bind to the M2R receptor on the adrenal gland. Our findings that the partial mRNA sequences of POMC, and the partial amino acid sequence of ACTH, did not differ between normal and PPID horses suggests that lack of ACTH bioactivity in horses may not be associated with a nucleotide polymorphism in the encoding gene. To determine this conclusively, future studies aimed at a detailed comparison of the full length POMC sequences from a large number of healthy horses and PPID affected ones is required. Combined with the clinical and laboratory evidence that treatment with a D2R-agonist normalizes endogenous ACTH levels, and the clinical signs of disease, our findings would suggest that the lack of ACTH bioactivity in PPID horses is more likely associated with post-translational modification of the nascent ACTH, downregulation of the M2R receptor on the adrenal gland, or some other yet unidentified mechanism. This requires additional research.

The second main contribution of this research lies in the identification and validation of dependable house-keeping genes in equine PPID pituitary studies. While candidate genes for other tissues of interest were identified in a restricted number of tissues [9], the lack of proper internal control genes raised a challenge. Multiple candidate genes were assessed for use as internal control genes in the equine pituitary gland. The results showing that both 18S rRNA and YWHAZ could be used as an internal control/house-keeping gene is significant because this information has not previously been reported. The reduced variability in the expression of the latter gene resulted in its subsequent use in our study. Although not a major focus of this research, the validation of these house-keeping genes is a very important and novel advancement in equine endocrinology.

The most important and third contribution of this research is the finding that POMC, PC1 and PC2 mRNA expression was elevated in the pituitary gland of PPID horses. Although only gene expression was studied here, this supports the mechanistic theory behind dopamine-deficient elevations in plasma ACTH concentrations extrapolated from murine studies [6]. While both PC1 and PC2 mRNA expression increased in PPID horses, our results did not show the differential upregulation noted in D2R deficient mice, wherein PC1 increased in activity by 4 to 5 times, whereas that of PC2 increased by only 2 to 3 times [6]. There are several possible explanations. Firstly, while mammalian hormone mechanisms are typically conserved, species specific differences do exist. Secondly, the murine studies that postulated a lack of dopamine, used type-2 dopamine receptor deficient D2R knock-out mice. These mice do not actually suffer from a true dopamine-deficiency. It is possible that differential receptor binding may have altered the relative upregulation of the prohormone convertase sub-types. Finally, our method of whole gland extraction precluded cellular localization of the PC enzymes. It is possible that differential upregulation occurred in multiple areas of the gland with the overall result that there was not a difference between PC1 and PC2. Had tissue been removed from specific PI or PD regions, it is possible that differential upregulation in the face of PPID may have been revealed.

There were no differences in the concentration of POMC within the plasma of cerebral blood collected from the ventral cavernous sinus between PPID horses and control horses. The lack of changes in circulating POMC, despite an increase in POMC mRNA expression, also suggests that post-translational or secretory defects are likely contributors of endocrine pathophysiology in equine PPID. It is likely that the protein would have been cleaved into the downstream, smaller peptides, including ACTH, α-MSH and CLIP before release into the ventral cavernous sinus. Our findings concur with previously reported literature in that there were significant elevations in ACTH and α-MSH in PPID horses compared to control horses [1,13–16].

Together, our results at the mRNA level provide a strong foundation for studying the molecular mechanisms of the endocrine defects in equine PPID. The series of studies reported here supported the hypothesis that PC1 and PC2 in the equine pituitary gland is upregulated in PPID. This is associated with an abnormal abundance of POMC derived proteins being released into the blood of the ventral cavernous sinus, and then into the blood of the systemic circulation. We only determined partial sequences of POMC, PC1 and PC2. In addition, tissue expression of these proteins was not determined. Despite these limitations, our results conclude a defect in ACTH precursor and its processing enzymes in PPID. Future research should focus on more in depth studies on the endocrine milieu in PPID, during various stages of PPID.
